# Budget impact analysis of cervical cancer screening in Portugal: comparison of cytology and primary HPV screening strategies

**DOI:** 10.1186/s12889-019-6536-4

**Published:** 2019-02-26

**Authors:** Angela Pista, Carlos Costa, Conceição Saldanha, José Alberto Fonseca Moutinho, José Maria Moutinho, Fernando Arrobas, Carlos Catalão, Jari Kempers

**Affiliations:** 1 Present address: National Institute of Health, National Reference Laboratory of Gastrointestinal Infections, Lisbon, Portugal; 2 Research Centre for Public Health, Public Health National School, New University of Lisbon, Lisbon, Portugal; 3 Cytology Department, Unilabs, Oporto, Portugal; 4 Health Sciences Faculty, Beira Interior University, Covilhã, Portugal; 5 Child and Women Health Department, Cova da Beira Hospital Center, Covilhã, Portugal; 6 Gynaecologic Oncology Department, CUF Hospital, Oporto, Portugal; 7 Datamedica, Biostatistics Services and Consulting, Lisbon, Portugal; 8 Roche Diagnostic Systems, Lisbon, Portugal; 9 Roche Diagnostics, Almere, the Netherlands

**Keywords:** Cervical cancer, Screening, HPV, HPV 16/18 genotyping, Cytology, Budget impact

## Abstract

**Background:**

Primary Human Papilloma Virus (HPV) testing is the currently recommended cervical cancer (CxCa) screening strategy by the Portuguese Society of Gynecology (SPG) clinical consensus. However, primary HPV testing has not yet been adopted by the Portuguese organized screening programs. This modelling study compares clinical benefits and costs of replacing the current practice, namely cytology with ASCUS HPV triage, with 2 comparative strategies: 1) HPV (pooled) test with cytology triage, or 2) HPV test with 16/18 genotyping and cytology triage, in organized CxCa screenings in Portugal.

**Methods:**

A budget impact model compares screening performance, clinical outcomes and budget impact of the 3 screening strategies. A hypothetical cohort of 2,078,039 Portuguese women aged 25–64 years old women is followed for two screening cycles. Screening intervals are 3 years for cytology and 5 years for the HPV strategies. Model inputs include epidemiological, test performance and medical cost data. Clinical impacts are assessed with the numbers of CIN2–3 and CxCa detected. Annual costs, budget impact and cost of detecting one CIN2+ were calculated from a public healthcare payer’s perspective.

**Results:**

HPV testing with HPV16/18 genotyping and cytology triage (comparator 2) shows the best clinical outcomes at the same cost as comparator 1 and is the most cost-effective CxCa screening strategy in the Portuguese context. Compared to screening with cytology, it would reduce annual CxCa incidence from 9.3 to 5.3 per 100,000, and CxCa mortality from 2.7 to 1.1 per 100,000. Further, it generates substantial cost savings by reducing the annual costs by €9.16 million (− 24%). The cost of detecting CIN2+ decreases from the current €15,845 to €12,795. On the other hand, HPV (pooled) test with cytology triage (comparator 1) reduces annual incidence of CxCa to 6.9 per 100,000 and CxCa mortality to 1.6 per 100,000, with a cost of €13,227 per CIN2+ detected with annual savings of €9.36 million (− 24%). The savings are mainly caused by increasing the length of routine screening intervals from three to five years.

**Conclusion:**

The results support current clinical recommendations to replace cytology with HPV with 16/18 genotyping with cytology triage as screening algorithm.

## Background

Cervical cancer (CxCa) is one of the most common cancers in women, with nearly 500,000 new cases being diagnosed each year worldwide [1]. Its prevalence represents relevant costs for patients, their families and countries. Insiga et al. [2] reported that 75% of CxCa-diagnosed women died before sixty and 25% before turning forty. Researchers also estimated that 29% of them would be professionally active in the year they died and, based on their salaries’ projections, represented a revenue loss of 1.3 Billion USD, a figure superior to the direct costs associated with CxCa in the US. The introduction of cervical cytology as a screening method in the mid-twentieth century contributed to a decrease in the rate of CxCa, but its low sensitivity for CIN2+ requires a frequent repetition of the secreening process [3]. As a result, there is need for more efficient and cost-effective screening methods [4]. Therefore, actual knowledge lead to the definition of new strategies of prevention and practice management that include Human Papillomavirus (HPV) testing and prophylactic vaccination. Portugal started the first organized cervical cancer screening program in the Centre Region in the late 90’s, extending it to more than half of the country nowadays. The HPV quadrivalent vaccine was introduced in the national vaccination program in 2008 extending the coverage to almost 90% of women. Regarding screening, the Portuguese Society of Gynaecology consensus document considers Pap Cytology with ASCUS HPV triage every three years as adequate. Nevertheless, also point out the primary high-risk HPV (hrHPV) testing with cytology triage every five years as the recommended screening algorithm [5], based on the superior sensitivity of the HPV assay, validated by prospective clinical trials. Ronco G et al. (2014) [3] point out that HPV-based screening provides 60–70% greater protection against invasive cervical carcinomas when compared to Pap cytology. Following this recommendation a law decret was published in 2017 confirming HPV as the primary screening test with 16/18 genotyping as a triage test for direct colposcopy and Pap cytology as a triage for other 12 hrHPV types [6], which is determining a change in the screening algorithm. Despite this fact, only some of the organized screening programs implemented the project. In this context, a comparison of the clinical and budget impact of different screening strategies will help to clarify the health care gains obtained with the adoption of the new screening algorithm. This is the first budget impact analysis to date on this subject evaluating the best scenarios for Portugal.

## Methods

### Study population

The modelling is carried out on a hypothetical national cohort of 2,078,039 25–64-year-old Portuguese women fitting the target age groups for cervical cancer screening. The same cohort was used for all the three screening strategies under comparison [7], assuming exclusion of ineligible patients, such as hysterectomized women. CxCa screening compliance rate, attendance at re-test and next routine screening were assumed to be 70.7% [8] and 85.0% [9], respectively.

### Compared screening strategies

**Fig. 1:**
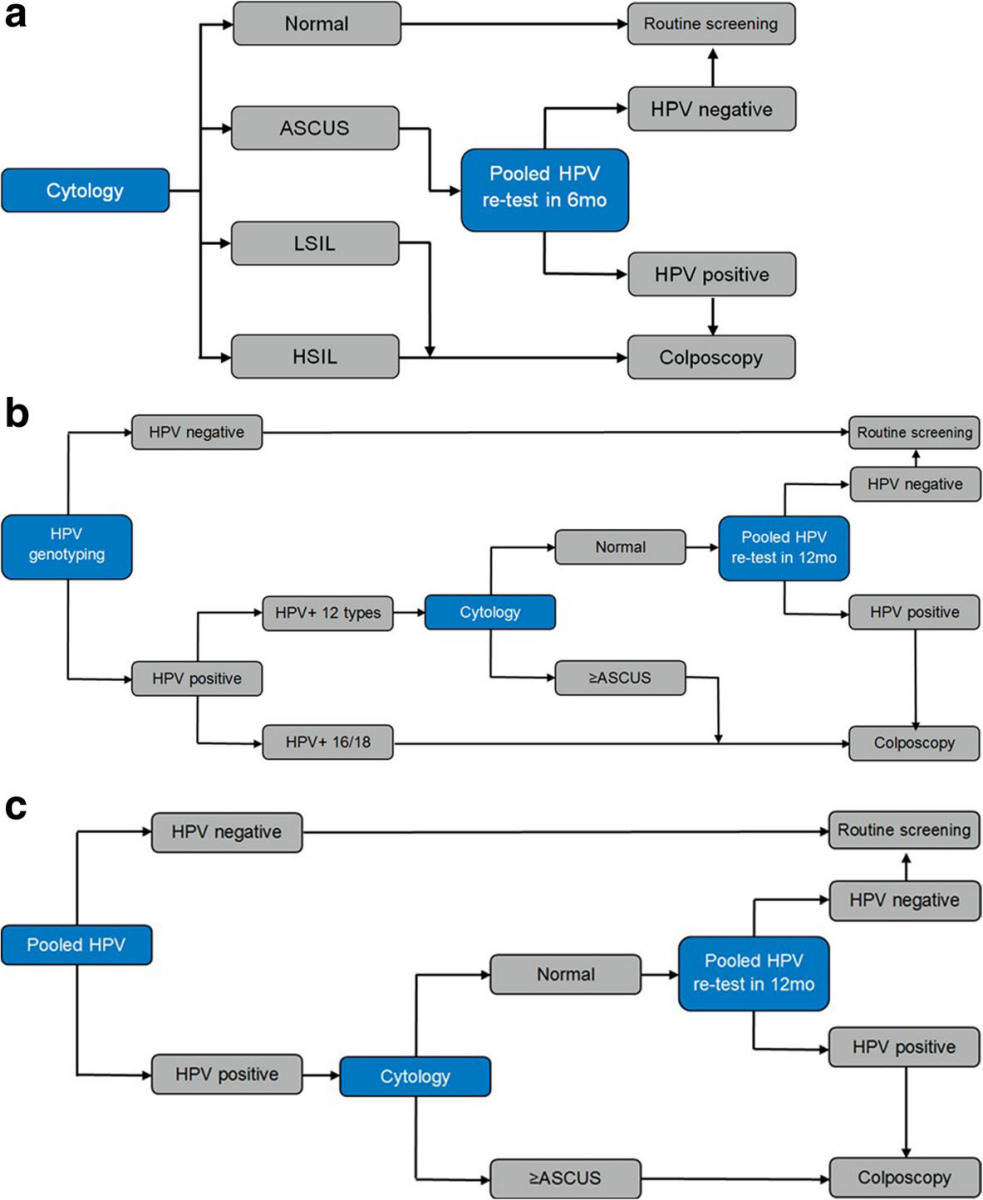
**a** The current practice cytology with pooled HPV triage. **b** Comparator 1 pooled HPV with reflex cytology triage. **c** Comparator 2 HPV test with genotyping and reflex cytology triage

The compared screening strategies in this evaluation were based on the Portuguese Society of Gynaecology consensus on cervical cancer screening [5]. Three primary CxCa screening strategies are compared. The current practice: cytology with ASCUS HPV triage every three years (Fig. 1-a). Women with normal cytology return to routine screening. Women with LSIL or worse are referred for colposcopy. ASCUS results are triaged with pooled HPV test within 6 months. HPV negative women return to routine screening and HPV-positive undergo colposcopy.

Comparator strategy 1: HPV (pooled) test with reflex cytology triage every five years (Fig. 1-b). HPV negative women return to routine screening. HPV positive results are triaged with reflex cytology. Women with cytology results ASCUS or worse are referred for colposcopy. Normal cytology results are followed up with HPV re-test in twelve months. Women with HPV-negative re-test return to routine screening and HPV-positive re-tests undergo colposcopy.

Comparator strategy 2: HPV test with 16/18 genotyping with reflex cytology triage every five years (Fig. 1-c). HPV negative women return to routine screening. Women with hrHPV genotypes 16/18 are directly referred for colposcopy. HPV genotypes other than 16/18 (HPV+ 12 types) are triaged with reflex cytology. Women with cytology results ASCUS or worse are referred to colposcopy. Normal cytology results are followed up with HPV re-test in twelve months. Women with HPV-negative re-test return to routine screening and HPV-positive re-tests undergo colposcopy.

### Model structure

**Fig. 2:**
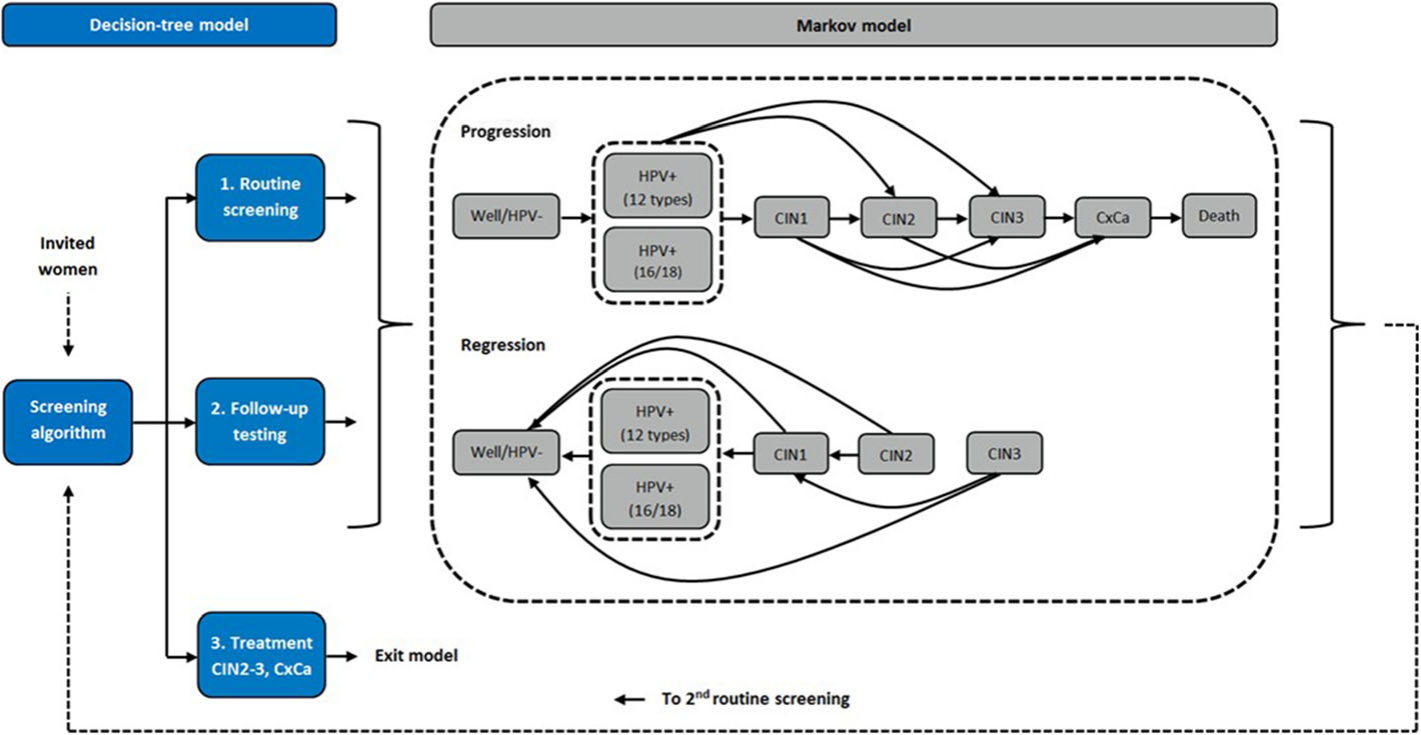
The model structure and patient flow

An Excel-based (Microsoft Office 365®) budget impact model was developed to evaluate screening performance, clinical outcomes and budget impacts of the CxCa screening strategies during two routine screening cycles. The model consists of two main components: a decision-tree model and a Markov model (Fig. 2). The decision-tree simulates the performance of the screening strategies. Women are divided into three groups according to their test results; 1) healthy, who return to routine screening, 2) those who require follow-up testing, and 3) diagnosed CIN2–3 or CxCa who are treated and exit the model. The groups 1 and 2 continue to the Markov model. The Markov model simulates natural history of HPV infection, CIN and CxCa in one-month cycles. During each cycle, women can remain on the same stage, progress to the next, or regress to the previous stage. The transition probabilities between the health stages are shown in Table 1 [10–24]. The model does not differentiate between the stages of CxCa and only includes the probability of dying from invasive CxCa, not considering mortality from other causes.

**Table 1:** Annual transition (progression / regression) probabilities of the Markov model; Natural history inputs for HPV infection, CIN, and cervical cancer

	Model input	References
Annual Progressio		
Well to hrHPV infection	4.2%	Kulasingam et al. (2011) [10]
Progression from hrHPV (12 types)		
to CIN1	8.1%	Kulasingam et al. (2011) [10]; Insinga RP et al. (2007) [11]
to CIN2	0.1%	Khan MJ et al. (2005) [12]
to CIN3	0.1%	
Progression from hrHPV 16/18		
to CIN1	9.9%	Insinga RP et al. (2007) [11]; Insinga RP et al. (2009) [13]
to CIN2	0.6%	Khan MJ et al. (2005) [12]
to CIN3	1.5%	
Progression from CIN1		
to CIN2	3.2%	Weighted average: Kataja V et al. (1989) [14]; Holowaty P et al. (1999) [15]; Matsumoto K et al. (2006) [16]; Omori M et al. (2007) [17]; Guedes AC et al. (2010) [18]
to CIN3	0.9%	
to CxCa	0.3%	
Progression from CIN2		
to CIN3	4.2%	Weighted average: Kataja V et al. (1989) [14]; Holowaty P et al. (1999) [15]; Matsumoto K et al. (2006) [16]; Omori M et al. (2007) [17]; Guedes AC et al. (2010) [18]
to CxCa	1.8%	
CIN3 to CxCa	3.4%	Weighted average: Kataja V et al. (1989) [14]; Holowaty P et al. (1999) [15]; McCredie et al. (2008) [19]
Annual mortality rate for CxCa	0.6%	SEER data. 5 year survival of 68% converted to annual mortality rate [20]
Annual Regression		
Regression from hrHPV (12 types)		
with normal cytology to well	58.6%	Bulkmans NJ et al. (2007) [21]
with borderline/mild cytology to well	45.6%	
Regression from hrHPV 16/18		
with normal cytology to well	43.8%	Bulkmans NJ et al. (2007) [21]
with borderline/mild cytology to well	21.8%	
Regression from CIN1		
to well	21.2%	Weighted average: Kataja V et al. (1989) [14]; Holowaty P et al. (1999) [15]; Matsumoto K et al. (2010) [22]
to hrHPV	2.4%	
Regression from CIN2		
to well	9.4%	Weighted average: Kataja V et al. (1989) [14]; Meyskens FL Jr. et al. (1994) [23]; Holowaty P et al. (1999) [15]; Matsumoto K et al. (2006) [16]; Omori M et al. (2007) [17]; Castle PE et al. (2009) [24]; Guedes AC et al. (2010) [18]
to CIN1	9.4%	
Regression from CIN3		
to well	3.9%	Weighted average: Kataja V et al. (1989) [14]; McCredie et al. (2008) [19]
to CIN1	1.6%	

### Model inputs

#### Epidemiology and test performance

The prevalence of HPV (10.5%) and HPV genotypes 16/18 (2.1%) among 25–64-year-old women are based on the CLEOPATRE epidemiological study in Portugal [25, 26]. The prevalence of CIN1 (1.4%), CIN2 (0.4%), CIN3 (0.6%) and CxCa (0.048%) are taken from the ATHENA trial [27]. The US-based trial assessed the performance of Thinprep® Liquid Based Cytology (Hologic) and the cobas® HPV test (Roche) in a cohort of 40,900 over 25-year-old women [28] was chosen because it is one of the largest studies until now to compare the performance of Pap cytology, HPV test and partial genotyping in a screening population. Additionally, data from Portugal on this matte is unavailable. The test performance inputs of Liquid Based Cytology (LBC) and HPV test (pooled and 16/18 genotyping) are also based on the ATHENA trial data (Table 2) [28]. Model inputs were based on the entire cohort and not stratified by age. Data for the natural history of cervical cancer was taken from the scientific literature (Table 3). Colposcopy is assumed 100% sensitive and specific.

**Table 2:** Prevalence of HPV, CIN and Cervical Cancer

Prevalence of hrHPV	8.4%	Pista et al. (2013) [26]
Prevalence of HPV16 and/or 18	2.4%	Pista et al. (2013) [26]
Prevalence of CIN1	1.4%	Wright et al. (2012) [27]
Prevalence of CIN2	0.4%	Wright et al. (2012) [27]
Prevalence of CIN3	0.6%	Wright et al. (2012) [27]
Prevalence of invasive cervical cancer	0.048%	Wright et al. (2012) [27]

**Table 3:** Test performance inputs of cytology and Cobas® HPV test (pooled and 16/18 genotyping)

Input	Value	Source
Cytology (threshold ASCUS)		
Sensitivity CIN2	52.6%	Castle et al. (2011) [28]
Sensitivity CIN3	52.8%	Castle et al. (2011) [28]
Sensitivity CxCa	52.8%	*assumed to be equivalent to CIN3*
Specificity CIN2+	76.1%	Castle et al. (2011) [28]
Cytology (threshold LSIL)		
Sensitivity CIN2	39.2%	Castle et al. (2011) [28]
Sensitivity CIN3	40.1%	Castle et al. (2011) [28]
Sensitivity CxCa	40.1%	*assumed to be equivalent to CIN3*
Specificity CIN2	86.5%	Castle et al. (2011) [28]
Cytology (threshold HSIL)		
Sensitivity CIN2	20.3%	Castle et al. (2011) [28]
Sensitivity CIN3	26.2%	Castle et al. (2011) [28]
Sensitivity CxCa	26.2%	*assumed to be equivalent to CIN3*
Specificity CIN2	98.3%	Castle et al. (2011) [28]
Cobas® HPV test (pooled)		
Sensitivity CIN2	88.2%	Castle et al. (2011) [28]
Sensitivity CIN3	92.0%	Castle et al. (2011) [28]
Sensitivity CxCa	92.0%	*assumed to be equivalent to CIN3*
Specificity CIN2	57.8%	Castle et al. (2011) [28]
Cobas® HPV test with genotyping 16/18		
Sensitivity 16/18 CIN2	51.8%	Castle et al. (2011) [28]
Sensitivity 16/18 CIN3	59.5%	Castle et al. (2011) [28]
Sensitivity 16/18 CxCa	65.3%	Castle et al. (2011) [28]
Specificity CIN2	75.3%	Castle et al. (2011) [28]

#### Costs

The costs are divided into three main categories; screening, diagnostic and treatment. Cost inputs are based on the Portuguese diagnosis-related group (DRG) prices in 2014 and on published data (Table 4) [29–31]. Screening costs include office visits, primary, triage and re-tests. Prices for the HPV test (pooled and 16/18 genotyping) are assumed to be the same as the DRG price of cytology (€27.40) because the current DRG price for the HPV test was set for a triage scenario and so it is too high for a primary screening test for CxCa [29]. Diagnosis costs relate to diagnostic consultations, colposcopies and biopsies. Treatment costs include CIN2–3 and CxCa treatment [30, 31]. The budget impacts are calculated from the healthcare provider’s perspective and presented in Euros.

**Table 4:** Medical costs used in the model

Parameter	Euro	References
SCREENING COSTS		
Office visit (routine/repeat screening)	31.00€	[29]
Cytology test (liquid-based)	27.40€	[29]
HPV test (pooled)	27.40€	Assumed to have the same price as for cytology test
HPV test with 16/18 genotyping	27.40€	Assumed to have the same price as for cytology test
DIAGNOSTIC COSTS		
Office visit (diagnostic follow-up)	31.00€	[29]
Colposcopy plus biopsy	34.40€	[29]
TREATMENT COSTS		
Treatment for cervical intraepithelial neoplasia (Grade ≥ 2)	907.57€	Adapted from: Costa C [30]; Santana R et al. [31]
Treatment for invasive cervical cancer	10423.29€	Adapted from: Costa C [30]; Santana R et al. [31]

### Model outputs

The performance of the screening strategies is assessed with the percentage of CIN2–3 and CxCa cases detected and with the number of colposcopies needed to detect one disease case (CIN2–3 and CxCa). The clinical impacts, in the screened population, are measured with an annual incidence of CxCa and annual CxCa mortality. The costs are presented as average annual costs during the two routine screening cycles. The annual costs are used to take into consideration the different routine screening intervals. Annual budget impacts of the primary HPV screening strategies are calculated against the current practice. Further, the costs outputs include the costs per screened woman and cost of detecting a disease (CIN2–3 and CxCa).

#### Sensitivity analysis

A sensitivity analysis was conducted to analyse whether clinical inputs from different published scientific literature sources [32–35], other than the primary reference used, would change the budget impact outputs of the model. A sensitivity analysis was also conducted to understand how the variation of the cost of the HPV test and LBC (half vs double) would affect the budget impact of the different screening strategies.

## Results

### Screening performance and clinical outcomes

**Fig. 3:**
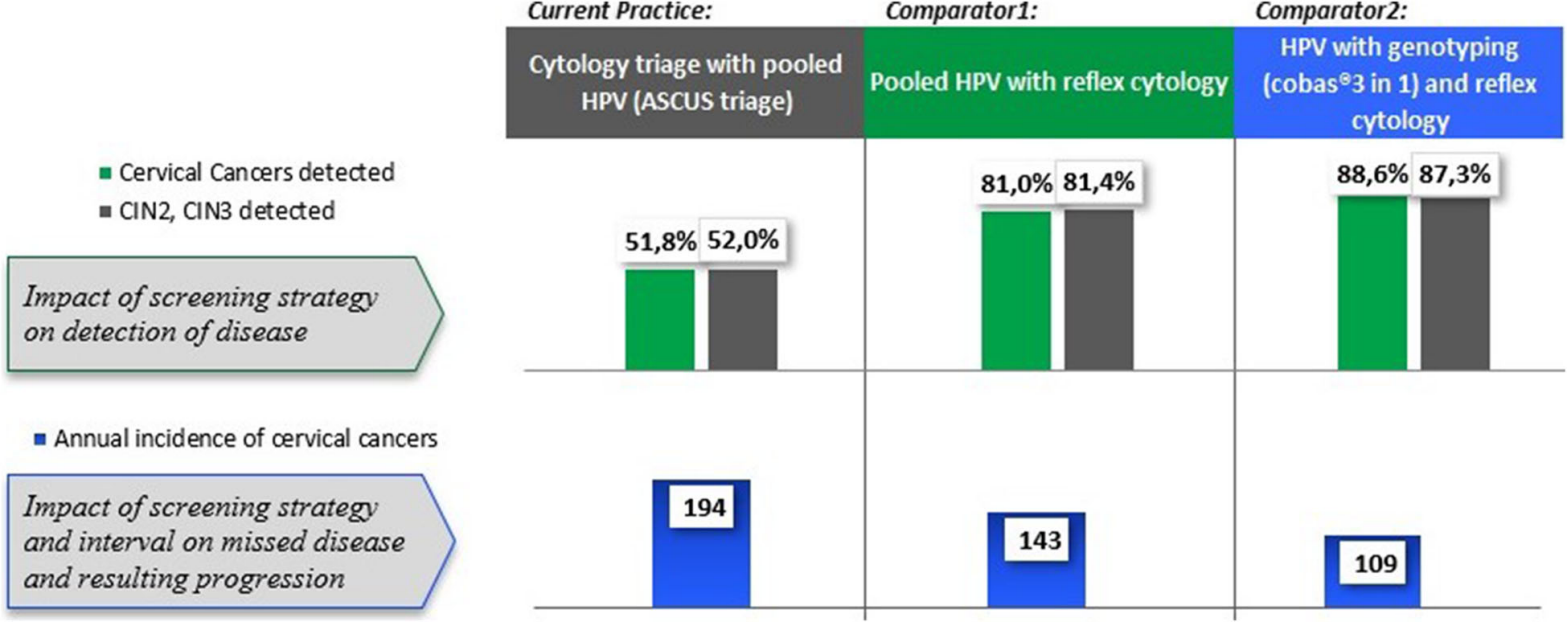
Performance of the screening strategies during two screening cycles. Detected cervical cancers and CIN2-CIN3 cases

According to the model results, the current cytology-based screening strategy detects 51.8% of CxCa and 51.9% of CIN2–3 cases. Comparator strategy 1: HPV (pooled) test with reflex cytology triage, increases the detection of CxCa to 81.0% and of CIN2–3 to 81.4%. Comparator strategy 2: HPV 16/18 genotyping with reflex cytology triage, improves the detection of CxCa to 88.6% and of CIN2–3 cases to 87.3% (Fig. 3). During the two screening cycles, the screening performances of the HPV-based comparator strategies 1 and 2 reduce annual incidence of CxCa in the screened population from 9.3 to 6.9 and 5.3 per 100,000, respectively. Furthermore, comparators 1 and 2 reduce annual CxCa mortality in the screened population from cytology-based screening from 2.7 to 1.6 and 1.1 per 100,000, respectively. This means avoiding an extra 51 CxCa deaths per year in comparator 1 and avoiding 85 extra CxCa deaths per year in comparator 2 for the simulated cohort. The number of colposcopies needed to detect a disease case (CIN2–3 and CxCa) also decrease from 10.1 to 8.2 in comparator 1 and 9.6 in comparator 2 (Table 5).

**Table 5:** Annual cervical cancer incidence and mortality in the screened population (1,764,000 25–65-year-old Portuguese women) and per 100,000. The number of colposcopies required per detected disease (CIN2–3 and CxCa)

	Current practice:		Comparator 1:		Comparator 2:	
	Cytology with pooled HPV triage		Pooled HPV test with reflex cytology triage		HPV genotyping with reflex cytology triage	
	Screened pop.	per 100,000	Screened pop.	per 100,000	Screened pop.	per 100,000
Annual CxCa incidence	194	9.3	143	6.9	109	5.3
Annual CxCa mortality	56	2.7	34	1.6	22	1.1
Colposcopies per disease (CIN2–2, CxCa) detected	10.1		8.2		9.6	

### Budget impact

**Fig. 4:**
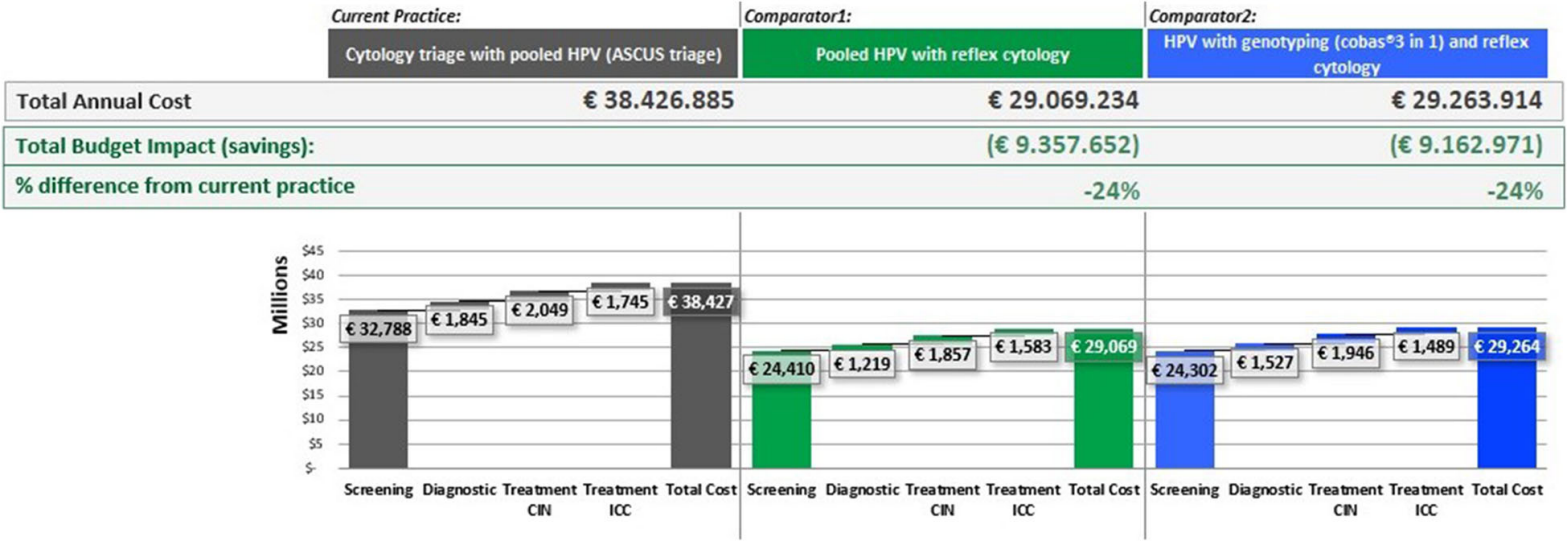
Total annual costs of the screening strategies. Budget impact in comparison to the current practice. A breakdown of annual costs per screening diagnostic

The average annual costs of the cytology-based strategy are €34,43 million. Annual costs of comparator 1 are €29,07 million and comparator 2 represent €29,26 million (Fig. 4). Both primary HPV screening strategies originate cost-savings. Comparator 1 decreases the total annual costs in €9,36 million (− 24%) and comparator 2 in €9,16 million (− 24%).

Figure 4 portrays a breakdown of annual costs of the three screening strategies. Screening costs are the largest component of all the three strategies. Annual screening costs (screening and triage tests and office visits) of the current practice and comparators 1 and 2 are €32,79 million, €24,41 million (− 25.6%) and €24,30 million (− 25.9%), respectively. Overall cost savings result from the reduction of screening costs. These cost savings are caused by the longer routine screening interval of the primary HPV screening strategies. Annual diagnosis costs (follow-up consultations, colposcopies and biopsies) of the current practice and comparators 1 and 2 are €1,85 million, €1,22 million (− 34.0%) and €1,53 million (− 17.0%), respectively. In both primary HPV screening strategies, the declining incidence of CIN2–3 and CxCa results in lower annual treatment costs. The treatment costs (CIN2–3 and CxCa combined) decline from €1,75 million to €1,58 million (− 9.7%) in comparator 1 and to €1,49 million (− 14.8%) in comparator 2. Average cost per screened women in the current practice and comparators 1 and 2 are €18,49, €13,99 and €14,08, respectively. In addition, the cost of detecting a disease case (CIN2–3 or CxCa) decreases from €15,845 to €13,227 in comparator 1 and to €12,795 in comparator 2.

### Sensitivity analysis

The sensitivity analysis shows that the comparator screening algorithms accuracy varies in a higher level than relative costs versus the current practice. Comparator 1 (Table 6) and 2 (Table 7) would reduce costs vs usual care if inputs from the POBASCAM and PavDag studies would be used for modelling instead of the ATHENA [32,33]. Modelling with an LBC sensitivity of 73.0% for pap cytology sensitivity in this model (highest pap cytology sensitivity for CIN2+ found in the ATHENA trial) [34], would improve the clinical performance of usual care, but would not alter the cost difference towards comparator strategies. On the other hand, cutting the cost of the HPV test by half (€13,7) would improve the savings vs usual care to 38.0% for comparators 1 and 2, while doubling the HPV test cost (€54,8) would still save 3.0% of the screening budget for Comparator 1 or 2.

**Table 6:** Clinical performance of triaging all hrHPV+ women with LBC

Study	Sensitivity (CIN2+)	Specificity (CIN2+)	Relative cost of Comparator 1
ATHENA (Castle PE et al., 2011 [28])	88.2%	57.8%	− 24%
POBASCAM (Dijkstra M et al., 2013 [32])	66%	81.4%	−26%
PavDag (Stanczuk GA. et al., 2017 [33])	68.3%	89.1%	− 26%

**Table 7:** Clinical performance of triaging hrHPV+ women with HPV16/18 genotyping and using LBC to triage OHR

Study	Sensitivity (CIN2+)	Specificity (CIN2+)	Relative cost of Comparator 2
ATHENA (Wright T et al., 2014 [27])	93.3%	62.8%	− 24%
ATHENA (Castle PE et al., 2011 [28])	51.8%	75.3%	− 24%
POBASCAM (Dijkstra M et al., 2013 [32])	90.3%	57.6%	−25%
PavDag (Stanczuk GA. et al., 2017 [33])	93.3%	52.7%	−24%

## Discussion

Overall, the output of the decision-tree and Markov model suggest the replacement of the current cytology-based screening with any of the two primary HPV screening strategies. It improves the detection of CIN2–3 and CxCa, displays better clinical outcomes and creates substantial cost-savings for the Portuguese healthcare system. The results are in line with similar health economic outcome research (HECON) studies [36–39] and support the current clinical consensus on the move towards molecular screening for cervical cancer not just in Portugal but also other European countries. Nevertheless, as this study is based on a computer-based model cohort, a randomized clinical trial or real-world studies with randomized samples and appropriate methodologies are needed as further research to generalize these results to the population.

The major goal of cervical cancer screening is to detect its precursor lesions and treat them before they become invasive. According to the findings of the meta-analysis of 4 European randomized trials (Swedescreen (Sweden) [40], POBASCAM (Netherlands) [41], ARTISTIC (England) [42] and NTCC (Italy) [43], reinforced by the recent findings of the COMPASS (Australia) [44] and FOCAL (Canada) [45]), HPV-based screening provides 60–70% greater protection against invasive cervical carcinomas compared with Pap cytology and that screening intervals may be extended to at least five years [3]. In the US, the Kaiser Permanente Northern California (analysing data from more than 330,000 women) [46] and the ATHENA trial (recruiting around 47,000 women) [28] confirmed the vantage of the HPV test over pap cytology as a primary screening method.

In this study, HPV 16/18 genotyping with cytology triage (comparator 2) provides better clinical impacts, while lowering the costs. Therefore, it is the most cost-effective CxCa screening strategy in the Portuguese context. Comparator 2 also reduces annual CxCa incidence and mortality and reduces the total annual screening costs in 24.0%, as well as the cost of detecting a CIN2+ in 19,2%. The screening performance of HPV (pooled) test with cytology triage (comparator 1) is lower when compared to comparator 2 in its detection rates but has similar costs. The improved clinical outcomes obtained through this HECON modelling result from an improved screening performance of both HPV strategies and an earlier detection of CIN2–3 and CxCa. On the other hand, the substantial cost savings are caused by the increased length of the routine screening intervals, allowed by the enhanced sensitivity and negative predictive value of HPV based screening. The performance of Pap cytology is low in the model, but consistent with data from an international meta-analysis showing a detection rate of 53,0% [47].

Concerning the triage of HPV positive women, the authors of this study acknowledge the fact that pap cytology performance as a triage marker is affected by an expected increase in sensitivity and a decrease in specificity [48, 49]. This means that the “real-life” sensitivity of the HPV based algorithms can be higher and specificity lower, positively impacting clinical results (CIN2+ detection) and increasing costs. To clarify this subject, a sensitivity analysis compared results from other studies that also addressed triage strategies for HPV positive women, such as POBASCAM [32], PavDag [33] and also from the ATHENA three years follow up results [34]. The conclusion was that the cost difference towards cytology-based screening remained higher, consolidating the main findings on the cost savings created by the change towards HPV test-based screening.

The model also projects a difference in clinical outcomes between comparator 1 and 2 strategies: 5.9% in sensitivity for the detection of CxCa. The model results suggest that if the individual tests are used in a certain strategy or algorithm then the number of detected cases may differ. Although such differences were never reported from clinical trials, they can be partially explained by the fact that in this simulation, in comparator 1 all hrHPV positive / NILM cases (including HPV16 and 18 positives) are deferred and that 15.0% of these will actually miss follow-up.

This study has certain limitations. As any modelling study, the results are influenced by the input parameters and structure of the model. The performances of LBC and the HPV test (pooled and 16/18 genotyping) are based on data from a trial in the United States, not on Portuguese data. The clinical results are sensitive to changes in prevalence of HPV, HPV genotypes 16/18, CIN2 and CIN3. Also, the referral rates to colposcopy were determined in this model by the prevalence of the infection by the 14 hrHPV types in the Portuguese epidemiological study and these may differ if prevalence changes, as it is expectable in a vaccinated population. Another model limitation is that performance data reflect the baseline test (test performance among the general population) and that the same test in a re-testing situation among a previously hrHPV+ population may be different. The authors acknowledge that the model should allow two test performance inputs - one at baseline and another for re-test situation among previously hrHPV+ women. While HPV vaccination programs impact the cost of screening programs as shown in recently published real world data [50], this model does not take that in consideration which may be considered also as a limitation.

The cost results are sensitive to changes in prices of cytology, HPV test and screening consultation. In the absence of a DRG reflecting the use of HPV as a screening test, the price of HPV tests was assumed the same as the cytology DRG. The future DRG price for an HPV test may be different. In this case, the authors tried to simulate the reality for Portugal as much as possible. In our country, there is no screening program at national level, and opportunistic and organized programs occur regionally with different levels of implementation. In the current setting, DRG prices are currently used as a reference for pricing both HPV and Cytology and the volume of tests would not allow for a cost reduction. A sensitive analysis varying the price of HPV test to either twice or half of that of cytology, shows that HPV based screening algorithms would remain cost-saving independently of the chosen scenario. Furthermore, the cost of screening can be lower if more suppliers compete in fair tenders. The guideline based on reproducibility and equivalent accuracy defined by Arbin et al. [51] is a milestone in HPV-based cervical cancer screening. There are currently 8 HPV DNA assays fully matching these criteria, which can be recommended for HPV-based cervical cancer screening using clinician-collected cervical samples, half of which offer partial, extended or full genotyping. This means the offer is large and competitive, making public tenders able to implement either of the Comparator strategies. This is important not just for Portugal, but also for other countries. The time-horizon of this modelling study is limited to two screening cycles. Policymakers would like to understand to which level CxCa incidence and mortality decrease, after several screening cycles.

## Conclusion

The results suggest that the replacement of the current screening practice based on cytology testing every three years to HPV primary screening with cytology triage every five years improves the detection of CIN2–3 and CxCa, provides better clinical outcomes and creates substantial cost-savings for the Portuguese healthcare system. These cost-savings are mainly generated by increasing the screening interval. Further triage of HPV positive women with partial 16/18 genotyping would improve the efficiency of the screening program. Therefore, the results of this HECON evaluation confirm the Cervical Cancer Screening recommendations of the Portuguese Society of Gynaecology [5] and the ongoing screening algorithm changes in the Portuguese organized screening programs. The results of this work also open directions for future research: a) the inputs used on this model could be refined with real world data coming from the organized screening programs, b) evaluation of the economic benefit of using emerging biomarkers like p16/ki-67 or methylation to further refine the triage of HPV+ women and c) re-model taking vaccine immunization into consideration.

## Abbreviations

ARTISTIC: A Randomized Trial In Screening To Improve Cytology
ATHENA: Addressing The Need for Advanced HPV Diagnostics
BIM: Budget Impact Model
CIN: Cervical Intraepithelial Neoplasia
CxCa: Cervical Cancer
DGR: Diagnosis-Related Groups
HPV: Human Papillomavirus
hrHPV: High-risk Human Papillomavirus
ISPOR: International Society for Pharmacoeconomics & Outcomes Research
LBC: Liquid Based Cytology
NTCC: New Technolog in Cervical Cancer
POBASCAM: Population Based Screening Study Amsterdam
SPG: Portuguese Society of Gyneacology
